# Statins as anti‐tumor agents: A paradigm for repurposed drugs

**DOI:** 10.1002/cnr2.2078

**Published:** 2024-05-06

**Authors:** Sneha Tripathi, Ekta Gupta, Sanjeev Galande

**Affiliations:** ^1^ Laboratory of Chromatin Biology & Epigenetics Indian Institute of Science Education and Research Pune India; ^2^ Centre of Excellence in Epigenetics, Department of Life Sciences Shiv Nadar Institution of Eminence Gautam Buddha Nagar India

**Keywords:** anti‐cancer drug, cholesterol, combinatorial therapy, drug repurposing, lipid metabolism, mevalonate pathway, statins

## Abstract

**Background:**

Statins, frequently prescribed medications, work by inhibiting the rate‐limiting enzyme HMG‐CoA reductase (HMGCR) in the mevalonate pathway to reduce cholesterol levels. Due to their multifaceted benefits, statins are being adapted for use as cost‐efficient, safe and effective anti‐cancer treatments. Several studies have shown that specific types of cancer are responsive to statin medications since they rely on the mevalonate pathway for their growth and survival.

**Recent Findings:**

Statin are a class of drugs known for their potent inhibition of cholesterol production and are typically prescribed to treat high cholesterol levels. Nevertheless, there is growing interest in repurposing statins for the treatment of malignant neoplastic diseases, often in conjunction with chemotherapy and radiotherapy. The mechanism behind statin treatment includes targeting apoptosis through the BCL2 signaling pathway, regulating the cell cycle via the p53‐YAP axis, and imparting epigenetic modulations by altering methylation patterns on CpG islands and histone acetylation by downregulating DNMTs and HDACs respectively. Notably, some studies have suggested a potential chemo‐preventive effect, as decreased occurrence of tumor relapse and enhanced survival rate were reported in patients undergoing long‐term statin therapy. However, the definitive endorsement of statin usage in cancer therapy hinges on population based clinical studies with larger patient cohorts and extended follow‐up periods.

**Conclusions:**

The potential of anti‐cancer properties of statins seems to reach beyond their influence on cholesterol production. Further investigations are necessary to uncover their effects on cancer promoting signaling pathways. Given their distinct attributes, statins might emerge as promising contenders in the fight against tumorigenesis, as they appear to enhance the efficacy and address the limitations of conventional cancer treatments.

## 1 INTRODUCTION

The Mevalonate pathway (MVA), also known as the cholesterol biosynthesis pathway, plays a crucial role in maintaining cellular metabolic homeostasis.^1^, ^2^ The first step of the pathway involves the conversion of three molecules of Acetyl CoA into 3‐Hydroxy 3‐methylglutaryl CoA (HMG CoA), followed by its transformation into mevalonate through the action of the enzyme HMG CoA reductase (HMGR). This particular enzymatic reaction serves as the rate‐limiting step in the cascade, subject to rigorous regulation, and governs the fate of the downstream responsive genes.^3^ In instances where cellular cholesterol levels are insufficient due to inadequate signaling, the mevalonate concentration serves as a direct signal to activate the master transcriptional regulator known as Sterol Regulatory Element Binding protein (SREBP). SREBP, in turn, orchestrates the upregulation of cholesterol‐responsive genes, including HMGR.^4^, ^5^

**FIGURE 1:**
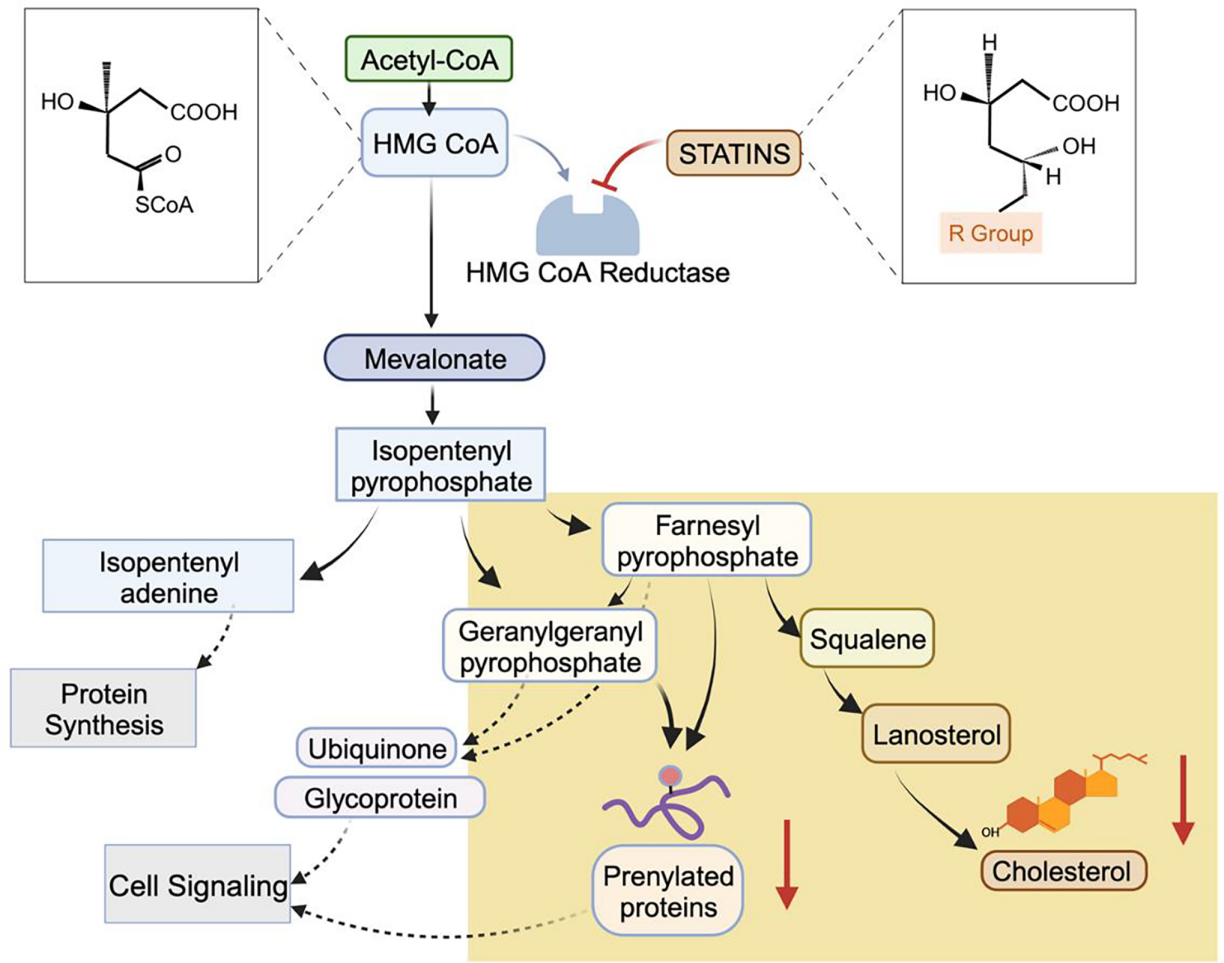
Schematic illustration of Mevalonate pathway (MVA) depicting effect of statins on the rate‐limiting step within the cascade.

The primary output of the mevalonate pathway is cholesterol (Figure 1), which plays a central role in maintaining the integrity of cell membranes. Nonetheless, the intermediate molecules that arise between mevalonate and cholesterol, such as squalene, lanosterol, and isopentenyl pyrophosphate, are involved in various biological processes.^6^, ^7^ An important consequence of this pathway is the post‐translational modification (PTM) known as isoprenylation, which occurs through the production of farnesyl pyrophosphate and geranylgeranyl pyrophosphate. These modifications are essential for regulating the activity of Rho and Ras family of GTPases, which are critical for signal transduction.^8^, ^9^ Furthermore, free fatty acids (FFAs), a product of the lipogenesis pathway, play a significant role not only in normal cellular signaling but also in dysregulated pathways leading to various disorders.^10^, ^11^, ^12^

Hypercholesterolemia is a dyslipidemic condition characterized by elevated levels of cholesterol in the bloodstream, which has abnormally high expression of the mevalonate pathway as one of the contributing factors.^13^ Over the years, pharmaceutical research has led to the establishment of well‐defined therapeutic approaches for this condition. The most potent class of drugs for its treatment is the statin family, which effectively inhibits the mevalonate pathway, resulting in efficient control of serum cholesterol levels.^14^ Statins are specifically designed to target the enzyme that catalyzes the rate‐limiting step of cholesterol biosynthesis, HMG‐CoA reductase. They achieve this through competitive inhibition, preventing the production of mevalonate itself. This compels cells to increase their uptake of free cholesterol from the bloodstream to meet their lipid metabolism requirements, ultimately leading to reduction in cholesterol levels in both blood and tissues.^15^, ^16^

In the 1970s, Arika Endo made the initial discovery of statins, identifying a compound derived from a fungal extract of *Penicillim citrinum*, and was labeled as compactin or mevastatin.^17^, ^18^ Encouraging results related to the specificity of inhibiting cholesterol biosynthesis primarily in the liver prompted clinical trials. Nevertheless, it was found that high or prolonged dosage led to complications. The upstream inhibition by statins and reduction in an important cellular component, cholesterol, accounted for its pleiotropic effects. Given that cholesterol and its derivatives are integral to normal cellular functions, inhibiting such an essential cellular building block was believed to affect other non‐target pathways, resulting in adverse side effects. Some of these side effects included gastrointestinal discomfort, inflammation, and even cataracts. However, during the 1980s, extensive research by Brown and Goldstein,^19^, ^20^ and further investigations in the 1990s,^21^ led to the conclusion that these side effects were not due to drug toxicity but were a result of its mechanism of action.^14^ Most of these effects were associated with how the drug was metabolized in the body, which depended on the *R* group attached to the lactone ring. This discovery motivated further research aimed at developing compounds with an intact lactone structure but with slight variations in the *R* group to improve the management of the drug.

It was determined that the *R* groups played a role in enhancing both drug delivery and the effectiveness of statins within the cells. Statins have been categorized into two types based on the *R* group attached to the lactone ring: hydrophobic and hydrophilic. Hydrophobic statins like simvastatin, fluvastatin, pitavastatin, lovastatin, and atorvastatin are known for their ability to easily penetrate the cells. While hydrophilic statins like rosuvastatin and pravastatin exhibit greater effectiveness after undergoing metabolism in the liver.^22^, ^23^ The first statin available for commercial use, lovastatin, was associated with side effects, such as nausea, fatigue and constipation. Subsequent iterations like simvastatin and pravastatin succeeded in reducing these complications. Moreover, synthetically produced statins like rosuvastatin and atorvastatin, demonstrated even more effective management of these side effects, attributed to modifications in the *R* group.^24^ Consequently, the significance of the *R* group is underscored by the increased number of novel and improved variants of statins in the market, which have shown better management of side effects compared to their early formulations.^25^

Beyond their role in addressing dyslipidemia, statins are utilized in treating conditions marked by elevated cholesterol levels as a prominent symptom, including coronary heart disease, atherosclerosis and diabetes mellitus. The potential applications of statins extended beyond cardiovascular diseases when correlations were established between various cancer types, including lymphoma,^26^ gastric cancers,^27^, ^28^, ^29^ breast cancer,^30^ and dyslipidemia.^15^ The role of cholesterol pathway gained increasing importance and underwent extensive investigation in tumorigenesis.^31^ It is now well‐established that tumor cells require the cholesterol biosynthesis pathway for their metabolic sustenance and rapid proliferation.^32^, ^33^ Cholesterol is not only essential for constructing cell membranes to accommodate the extensive multiplication of tumor cells but also plays a crucial role in cellular signaling in conjunction with other lipids. This includes free fatty acids (FFAs), oxysterols, prostaglandins, lipid modified oncogenic proteins, all of which play a vital role in tumorigenesis.^34^, ^35^, ^36^ Therefore, the exploration of using statins to prevent tumor progression by targeting the mevalonate pathway has become a subject of accelerated research.

One of the most groundbreaking and transformative proposition in recent times revolves around the use of statins as anti‐neoplastic agents. Numerous studies have unveiled a favorable correlation between statin use and improved prognosis in various cancer types. The increasing body of evidence underscoring the potential anti‐cancer attributes of statins primarily focuses on their mechanism of action in inhibiting tumorigenesis. As a result, statins are increasingly emerging as robust contenders for repurposing in the realm of cancer therapeutics.

In the initial step of the cascade, HMG CoA is generated by combining three Acetyl CoA molecules, subsequently being converted into mevalonate by HMG CoA reductase. The MVA pathway can be divided into three primary branches, with mevalonate as the central product, leading to the formation of Isopentenyl pyrophosphate that further acts as a precursor for several critical molecules. One branch (on the left) contributes to protein synthesis via Isopentenyl adenine as an important intermediate contributing toward translation of peptide through modified tRNAs, as well as isopentenylated nucleosides. Another branch (in the middle) generates essential biomolecules for cell signaling, such as ubiquinone, glycoproteins and prenylated proteins. The precursors for the two sub‐branches, responsible for the formation of ubiquinone and glycoproteins, are farnesyl pyrophosphate (FPP) and geranylgeranyl pyrophosphate (GGPP). The other sub‐branch originating from FPP and GGPP is involved in post‐translational modifications of proteins. The third branch (on the right) is the most extensively studied, as it is responsible for cholesterol production, involving intermediary molecules like squalene and lanosterol. Statins act as competitive inhibitors of HMG CoA reductase, possessing a structural resemblance to its substrate HMG CoA and competes for the enzyme's active site. The drug's lactone structure blocks the enzyme's function, leading to cessation of mevalonate production, while the specific *R* group varies according to the type of statin. Statins intervene at an upstream level, targeting the rate‐limiting step within the cascade, significantly influencing the downstream outcome. The primary consequence of this inhibition is a substantial reduction in cholesterol production, along with the downregulation of isoprenylation levels, which in turn affects the stability of target proteins.

## 2 REPURPOSING OF STATINS FOR CANCER THERAPEUTICS

Cancer remains a significant burden among various medical conditions, yet it exhibits the lowest success rate in clinical trials. Global data suggests that by 2025, more than 20 million individuals will be diagnosed with cancer.^37^ Notably, colon, prostate, and breast cancer, often incurable in their advanced stages with current treatments, will substantially contribute to this increase.^38^ To address both the current and future challenges, more effective cancer therapies are required. One approach being adopted in this direction is the repurposing of drugs already included in treatment protocols.^39^ The rationale behind using repurposed drugs in cancer therapeutics is grounded in the understanding that the physiological pathways they target are known to be dysregulated in tumorigenesis.^28^, ^40^, ^41^ As a result, these repurposed drugs should have the potential to impede tumor progression by targeting the altered physiological state of the cancer cells. Moreover, they can be considered for use in combination with existing treatment protocols to curb the advancement of cancer through its stages.

### 2.1 The paradigm of repurposing drugs

The concept of repurposing drugs, also known as rechanneling or repositioning, in pharmacology is being employed as an alternative approach to meet the demand for more potent, novel, and cost‐effective anti‐cancer medications.^42^ This strategy capitalizes on drugs that have multiple targets either off‐target or on‐target effects, allowing them to offer various modes of action. Repurposing offers a compelling advantage by providing patients with faster access to FDA‐approved medications that already have a well‐established safety and toxicity profile.^43^ Pre‐clinical research, clinical trials, and observational studies have accumulated evidence indicating the anti‐tumor efficacy of a wide‐ array of FDA‐approved compounds from therapeutic classes unrelated to cancer.^44^ These repurposed drugs, including cimetidine, clarithromycin, mebendazole and others are classified as highly potent for oncological treatment.^45^ They can inhibit tumor growth by triggering apoptosis or by directly or indirectly influencing the hallmarks of cancer. For instance, metformin, based on several epidemiological studies involving diabetes patients, has been suggested to exert a tumor‐protective effect.^46^

The process of drug repurposing in cancer encompasses multiple steps aimed at screening for potential anti‐neoplastic effects. A few important ways to identify drugs for repurposing include examining scientific databases to assess the off‐target effects of known drugs (referred to as knowledge mining), conducting in silico and in vitro experiments to evaluate cytotoxicity, engaging in animal studies, observing clinical cases, carrying out epidemiological investigations, and adopting a two‐pronged drug development approach which involves identifying specific mutations in DNA.^47^ These processes collectively help in the identification of potential candidates for repurposing of drugs in the field of cancer therapeutics. Despite the numerous benefits of medicinal repurposing, repositioning existing pharmaceuticals does present several challenges that must be addressed before they can be effectively employed in clinical trials. Given the current emphasis on targeted therapy, very few repurposed medications, such as thalidomide derivative compounds^39^ have been developed that directly and selectively affect the cancer cells. In contrast, the majority of prospective repurposed anti‐cancer drugs target the tumor microenvironment (TME).^48^ The purportedly repurposed anti‐cancer drugs reportedly influence the tumor microenvironment (TME), primarily by targeting the metabolic and immune components of the microenvironment. Five promising candidates for drug‐repurposing — namely, aspirin, celecoxib, β‐adrenergic antagonist, metformin, and statin — have garnered attention for their reported effects on TME, exhibiting anti‐tumor efficacy in preclinical models.^49^ For instance, in HNSCC mice, simvastatin induces metabolic reprogramming, reducing lactate generation and enhancing cancer susceptibility to MCT1 inhibitors.^50^ Additionally, statins may impact the immune microenvironment through modulation of immune checkpoints, cytokines or chemokines. In primary squamous lung cancer, statins inhibit CCL3 secretion by cancer cells and IL‐6 and CCL2 production by mesenchymal stromal cells (MSCs).^51^ Despite the potential of statins for repurposing in targeting the immune environment and suppressing lung cancer cells survival, a notable challenge in this approach arises from the possibility of off‐target effects and unintended immunomodulatory outcomes.^52^ Nevertheless, collaborative efforts involving clinicians, researchers, and pharmaceutical companies are actively exploring the implications of drug repurposing, with a steadfast commitment to ensuring patient well‐being.

### 2.2 How statins fit the paradigm

Statins possess various pleiotropic effects beyond their cholesterol‐lowering ability.^15^, ^21^ Notably, their potential as repurposed drugs for cancer treatment has garnered considerable attention due to their anti‐cancer properties.^53^ While numerous clinical and epidemiological studies have described statin's anti‐cancer attributes, the evidence supporting their effectiveness as anti‐cancer agents remains conflicting. It appears that specific molecular subtypes of cancer may respond differently to statin therapy, making it a context‐dependent phenomenon. There have been documented instances of widely varying responses to statin exposure among different cancer cell lines. For instance, in breast cancer, the sensitivity of statins has been associated with the presence or absence of the estrogen receptor (ER), with ER‐negative breast cancer cells displaying higher susceptibility to statin exposure.^54^ Clinical evidence suggesting increased tumor cell apoptosis following fluvastatin treatment in women with ER‐negative breast cancer reinforces these pre‐clinical findings.^55^ Independent research has indicated that tumor cells from diverse origins exhibit greater susceptibility, particularly those expressing higher levels of mesenchymal cells markers, such as vimentin.^56^, ^57^, ^58^ Furthermore, statins have been shown to selectively target cells undergoing epithelial‐to‐mesenchymal transition, suggesting their potential utility in limiting metastatic disease.^58^ While it remains unclear whether the mesenchymal characteristics of ER‐negative breast cancers contribute to their higher susceptibility to statins, the mechanism underlying the susceptibility of mesenchymal cancer cells to HMGCR suppression is not yet fully understood. Nevertheless, these findings lend support to the idea that the sensitivity to statins can be stratified based on tumor subtype. The question of whether statins yield clinical anti‐tumor effects is still under investigation. However, statins appear to enhance the efficacy of conventional cancer treatments and address their limitations, suggesting that they should be considered in the context of cancer‐specific combination therapy. One of the frequently used anti‐cancer medication Cisplatin, employed for a variety of malignant tumors, is associated with approximately 50% of treated individuals experiencing irreversible hearing loss.^59^, ^60^ Notably, Atorvastatin has a significant reduction in the frequency and severity of cisplatin‐induced hearing loss, thereby mitigating ototoxicity.^61^ However, to comprehensively understand the molecular pathways through which statins alleviate these drawbacks and side effects, further research is warranted. Additionally, growing body of evidence indicates that lipids,^62^ dietary factors,^13^, ^29^, ^63^ and obesity^64^, ^65^, ^66^ play direct roles in the development of various malignant tumors, often leading to metastatic disease and resistance to treatment.^67^, ^68^ Consequently, the use of statins as an adjuvant in anti‐neoplastic treatment regimens may render aggressive malignant tumors more susceptible.

## 3 ROLE OF STATINS IN ANTI‐CANCER TREATMENT AND POSSIBLE MOLECULAR MECHANISMS

### 3.1 A compilation of reported anti‐neoplastic effects mediated by statins

Numerous retrospective studies have highlighted associations between statins use and reduced cancer risk, lower cancer grades, and earlier stage diagnoses.^69^ These observations suggest that statins, by influencing the population of cancer stem cells, possess the potential to diminish metastatic tendencies, impede epithelial‐to‐mesenchymal transition, and reduce the likelihood of tumor recurrence.^58^, ^70^, ^71^, ^72^ For example, Simvastatin induces apoptosis in poorly differentiated cancer cells and dramatically suppresses proliferation through non‐canonical regulation of Rho GTPases and activating JNK pathway.^73^ Moreover, Simvastatin exhibits a capability to restrain the growth of aggressive tumors more effectively than differentiated mature cells, as observed in HCT116 cells and MCF‐7, Bcl2, and Erb2 overexpressing cells.^74^ Comparable correlations between efficacy and tumor grading has been noted for other statins, as observed in mouse embryonic stem cells, and Simvastatin has been shown to suppress self‐renewal by inhibiting Rho geranylgeranylation.^75^

**FIGURE 2:**
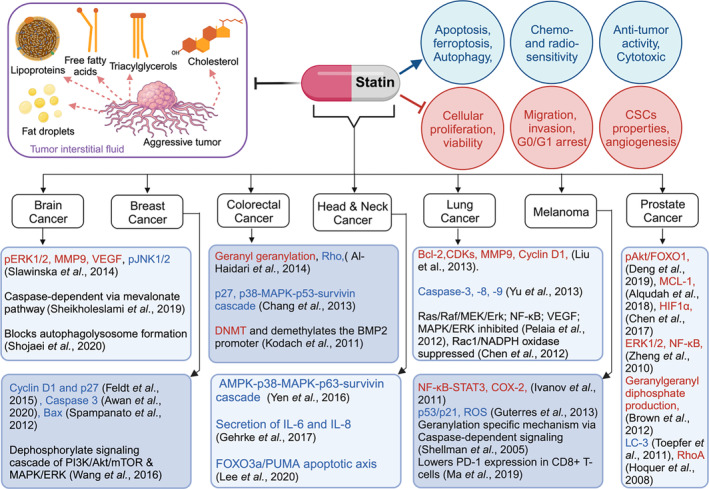
Role of statin in anti‐cancer treatment and possible molecular mechanisms.

The anti‐proliferative and apoptotic effects of statin have been examined in vitro in various cancer cell types including brain tumors,^76^ hepatic cancer,^77^ breast cancer,^78^ gastric cancer,^79^ colorectal cancer,^80^ lung cancer,^81^ and thyroid cancer.^53^, ^82^, ^83^ Statins are thought to act through distinct mechanistic pathways in different cancer types, modulating the cell cycle,^84^ apoptosis^85^ and enhancing sensitivity to chemotherapeutic agents^24^ as illustrated in Figure 2. Reports have suggested that simvastatin may increase the responsiveness of C26 mice colon cancer cells to 5‐FU therapy.^86^ Statins have also demonstrated the ability to transiently alter the levels of the prostate‐specific membrane antigen (PSMA) and the epidermal growth factor receptor (EGFR) on the surface of tumor cells, thereby augmenting the tumor‐binding avidity of monoclonal antibodies such as panitumumab, cetuximab, and huJ591, synergizing with their anti‐tumor effects.^86^ Furthermore, lovastatin has been observed to increase the sensitivity of gallbladder cancer to cisplatin.^80^ Available pre‐clinical evidence supports the concurrent use of statins and trastuzumab in reducing the cardiotoxicity of trastuzumab‐based therapy for HER2‐positive breast tumors.^68^

Statins have been found to reduce radiation resistance in head and neck malignancies by inhibiting the mevalonate pathway, indicating that this pathway could be a crucial target in combating the emergence of resistance.^87^, ^88^ Notably, the effect of statins on different types of tumors in various cancers types is likely influenced by the variation in cholesterol metabolism across different tissues. There exist notable structural differences in cholesterol production pathways among various tissues,^89^, ^90^ and the underlying reasons for why cells employing distinct pathways for cholesterol production remain not fully elucidated. Two primary pathways through which cholesterol contributes to cancer initiation are described in the literature.^91^, ^92^ The first emphasizes cholesterol's crucial role in cell adhesion and signaling processes, essential for normal cell functioning. The second underscores cholesterol's function as a precursor in the synthesis of sex hormones and other isoprenoid intermediates, linked to the development of specific cancer types. Given that not all cancer cell lines respond uniformly to statins, the sensitivity of their anti‐cancer effects varies depending on the tumor type. In a specific study, atorvastatin was assessed in two cell lines from seven distinct solid tumor types, including melanoma (SK‐MEL‐5, MDA‐MB‐435), lung (HOP‐92, NCI‐H322M), breast (HS‐578T, T47D), prostate (PC‐3, DU‐145), colon (HCT‐116, KM‐12), ovarian (IGROV1, OVCAR3), and brain (SF‐295, SF‐539) malignancies.^93^ Notably, different tumor cell lines exhibited diverse responses to atorvastatin treatment, with some displaying complete or partial suppression of growth, while other showed no sensitivity to the drug. A clinical trial conducted between 2001 and 2014 involving 9135 subjects with Hepatitis C virus (HCV) infection used fluvastatin and atorvastatin.^94^ The results demonstrates that statin use decreased the risk of fibrosis progression and led to a 49% reduction in hepatocellular carcinoma incidence. As a result, current research supports the notion that statins align with the paradigm of re‐purposed drugs that serve the dual purpose of lowering cholesterol while exerting anti‐cancer effects.

Within the tumor microenvironment, a variety of lipids, including free fatty acids, triacylglycerols, cholesterol, fat droplets and lipoproteins promote tumor growth and metastasis. Statin class of drugs could be repurposed and used in combination with chemotherapy and/or radiotherapy to target lipid metabolism due to their recognized anti‐cancer effects, distinct from their role in cholesterol regulation. The effect on molecular mechanisms can differ depending on the specific type of cancer and its stage. Changes in molecular players, denoted by downregulated (red) or upregulated (blue), result in diverse cellular effects in specific cancer types. For instance, cellular proliferation is inhibited through effects on signaling pathways such as PI3K/Akt,^95^ Erk 1/2,^96^, ^97^ Ras‐MAPK,^98^ NF‐κB^99^ and mTOR.^95^ Similarly, statin treatment can lead to either an increase or decrease in cell‐cycle regulators, including cyclin D1, p27, p53/p21,^100^, ^101^ p63‐survivin axis, and cyclin‐dependent kinases (CDKs) causing cell cycle arrest. Moreover, it activates pathways including caspase‐8/9,^102^, ^103^ Microtubule‐associated protein 1A/1B‐light chain 3 (LC3) for autophagy,^104^, ^105^ ferroptosis^106^ and apoptosis.^85^, ^107^, ^108^, ^109^, ^110^ Furthermore, statins inhibit angiogenesis^111^ and metastasis,^112^ reduce cancer stem cell markers by regulating metalloproteinase‐9 (MMP9),^113^ vascular endothelial growth factor (VEGF),^114^, ^115^ and the delocalization of Rho family proteins,^116^ as well as interleukins IL‐6, IL‐8.^117^ Statins are also reported to downregulate co‐inhibitory receptors like PD‐1, making them potential candidates for combination with immunotherapy.^118^ Additionally, they may increase the sensitivity of cells to chemotherapy and enhance their chemopreventive activity. Statins inhibit DNA methyltransferases (DNMTs) and activate bone morphogenetic protein (BMP) signaling pathway by demethylating the *BMP2* promoter, resulting in differentiation of cells and reduction in stemness potential.^119^

### 3.2 Exploring the molecular mechanisms of anti‐neoplastic effects of statins

The effectiveness of statins can be exhibited either in a cholesterol‐dependent manner or through non‐canonical effects.^120^ As statins lead to decreased levels of cholesterol, their action in cancer may result in compromised functionality of tumor cell membranes, thereby impeding tumor growth and metastasis.^121^ A recent study proposed cholesterol metabolism as therapeutic target in leukemia, highlighting the impact of atorvastatin on the RORγ/SREBP2 axis.^122^ Moreover, in cholesterol‐rich membranes, statins may undergo partitioning into rafts and forming complexes with specific proteins.^123^ Lipid rafts serve as signaling hubs facilitate signal transduction, partly mediated by caveolin‐1 (CAV1). Elevated expression of CAV1 is directly associated with a poor prognosis in cancer, as it can modulate the metastatic and invasive potential of malignant cells. By disrupting caveolae and lipid rafts, statin can influence cell death pathways in various types of cancer.^124^, ^125^

The role of cholesterol in lipid rafts and caveolae within the plasma membrane can influence proliferation and survival of cancer cells through its effects on signaling by receptors such as HER2,^126^ EGFR^127^ and CXCR4,^128^ as well as transducers and effectors, such as PI3K,^129^ SRC family kinases,^130^ and other regulators.^131^ Reduced cholesterol levels have the potential to disrupt immune cell rafts and caveolae, dispersing transducers and receptors that drive inflammatory signaling in both innate and adaptive immune cells.^132^, ^133^ A potential mechanism for the beneficial effects of statins may involve disruption of lipid rafts, leading to the inhibition of the PIK3/Akt signaling pathway, resulting in radiosensitization, as observed in HNSCC.^134^ Conversely, statins might exert some of their actions in a cholesterol‐independent manner. In addition to lowering cholesterol, statin mediated inhibition of mevalonate biosynthesis reduces the amount of isoprenoids formed, which in turn decreases geranylgeranylation and farnesylation of proteins, including small GTPases and other modified proteins.^135^ Statins have been demonstrated to restrict the synthesis of heme A in cytochrome C oxidase and ubiquinone (coenzyme Q10), potentially compromising the function of the mitochondrial electron transport chain.^120^ A major non‐canonical effect of statins involves the physiological consequences of reduced protein prenylation, which can profoundly affect cancer cells.^120^ Furthermore, statins' effect on isoprenoid production can result in a variety of pleiotropic outcomes. Since cell growth, survival, and differentiation rely on intracellular signaling proteins such as Ras, Rac, and Rho, their isoprenylated forms serve as lipid anchors and crucial signaling intermediates.^53^ In tumor cells, statins inhibit the isoprenylation, affecting both geranylgeranylation and farnesylation, thereby influencing oncogenic pathways. Consequently, it has been reported that statins may enhance anti‐tumor effects in combination treatments, as observed in EGFR‐targeted therapies.^136^, ^137^, ^138^ The growing body of population‐based research and clinical trials continues to bolster the multifaceted anti‐cancer effects of statins (Table 1).

**TABLE 1:** **Molecular pathways affected upon statin treatment.** The table summarizes characterized molecular pathways and mode of action of statins in various cancer types following in vitro, in vivo and clinical trial studies.

Cancer Type	Type of study	Statin/combination	Anti‐cancer mode of action	Reference number
**Breast**	in vitro	Simvastatin	Dephosphorylates signaling cascade of PI3K/Akt/mTOR and MAPK/ERK and cell cycle arrest in G1 phase	95
	in vitro and in vivo	Mevastatin + Histone deacetylase inhibitors	Inhibits autophagic flux by preventing Vps34/Beclin 1 complex formation and downregulating prenylated Rab7	162
	in vitro and in vivo	Simvastatin	Induces activation of PTEN expression via NF‐κB to inhibit breast cancer cell growth	163
	in vivo	Simvastatin + Doxorubicin	Down‐regulation of the cell cycle or induction of apoptosis	164
	Clinical trial	Fluvastatin	Inhibitory effect on proliferation and promotes apoptosis in women diagnosed with high‐grade breast cancer.	55
**Colorectal**	in vitro	Simvastatin	Induces the apoptosis of human colon cancer cells and inhibits IGF‐1‐induced ERK and Akt expression	165
	in vitro	Simvastatin, Fluvastatin and Atorvastatin	Induced p27^KIP1^ expression by downregulation of histone methyltransferase enhancer of EZH2	166
	in vitro and in vivo	Simvastatin	Inhibits geranyl geranylation, RhoA activation, p38 MAPK‐p53‐ survivin cascade activation	98, 111
	in vitro and in vivo	Atorvastatin +Nobiletin	Induced extensive cell cycle arrest and apoptosis in colon cancer cells, and enhanced chemopreventive activities against colon carcinogenesis in rats	167
	Clinical trial	Simvastatin +FOLFIRI	Tumor cell senescence, anti‐angiogenesis, and apoptosis.	168
**Brain**	in vitro	Simvastatin	Caspase dependent via mevalonate pathway	102
	in vitro	Simvastatin +Temozolomide	Inhibited the autophagy flux by blocking autophagolysosome formation	104
	Clinical trial	Thalidomide alternating with Fluvastatin and combined with Carboplatin and Vincristine	Significant reduction in tumor volume and a significant increase in survival of patients	169
**Head and neck**	in vitro	Lovastatin	Activates AMPK‐p38‐MAPk‐p63‐survivin cascade, activates apoptotic activity	170
	in vitro	Atorvastatin	Inhibits RhoC activity by reducing the phosphorylation of ERK1/2 and STAT3	139
	in vivo	Simvastatin +Monocarboxylate transporter 1 inhibitor	Reduces lactate production and promotes cancer sensitivity to MCT1 inhibitor	50
**Lung**	in vitro and in vivo	Simvastatin	Reduces proliferation and prevents osteolytic bone metastases of lung adenocarcinoma cells by regulating CD44, p53,MMP family and inactivates MAPK/ERK signaling pathway	113
	in vitro and in vivo	Simvastatin	Disrupts growth and survival in human SCLC cells by inhibiting Ras signaling	171
	Clinical trial	Simvastatin	Enhances the efficacy of gefitinib in a subgroup of gefitinib resistant NSCLC patients	172
**Melanoma**	in vitro	Simvastatin	p53/p21 pathway activation, intracellular ROS production	101
	in vitro	Pitavastatin + Dacarbazine	Activates apoptosis and autophagy resulting in synergistic cytotoxicity in melanoma cells	173
	in vivo	Simvastatin	Reduces PD‐1 expression in CD8 + T cells and effectively restores antitumor activity	118
	Clinical trial	Thalidomide, Dexamethasone and Lovastatin	Prolongation of overall survival and progression‐free survival	174
**Prostate**	in vitro	Atorvastatin, Mevastatin, Simvastatin and Rosuvastatin	Reduces migratory potential and colony formation of PC‐3 cells by inhibiting GGPP production.	112
	in vitro	Simvastatin	Inhibits Akt and reduced prostate‐specific antigen expression	175
	in vitro and in vivo	Atorvastatin + Celecoxib	Inhibits growth and activates Akt/ERK1/2, NF‐Kb pathway, cell growth arrest in G1 phase	97
	Clinical trial	Simvastatin+ Enzalutamide	Significant inhibition of enzalutamide‐resistant prostate cancer cell growth.	176

In the context of Rho protein functions in HNSCC proliferation and survival, in‐vitro studies have indicated that atorvastatin inhibits RhoC activity by reducing the phosphorylation of ERK1/2 and STAT3.^139^ This, in turn, results in decreased colony formation, invasion, and motility of cells.^139^ Simvastatin has similarly been shown to decrease cell proliferation, limit stress fiber production, and downregulate integrin β‐1.^140^ Moreover, simvastatin upregulates the cell cycle regulators p21 and p27.^140^ In the treatment of prostate cancer with lovastatin or simvastatin, RhoA is inactivated, triggering apoptosis in cancer cells and arresting the cell cycle in the G1 phase.^116^ Given that RhoA's pro‐proliferative and anti‐apoptotic effects are mediated by ERK1/2 activation, as documented previously,^141^, ^142^ statin therapy may reduce mTOR phosphorylation and ERK1/2 levels. Consequently, apoptosis may occur due to an increase in Bim expression.^143^ This concept finds support in the demonstrated statin‐induced apoptosis in human colon cancer xenografts and cells, as well as breast cancer.^144^, ^145^

The increased malignancy of cancer cells is attributed to cellular plasticity, facilitating adaptability to stress, invasion of their surroundings, and metastatic spread.^146^ Fluvastatin induces significant alterations the the morphology of pancreatic cancer cells, inhibiting their ability to spread in a dose‐dependent manner.^71^ Similar effects have been observed in prostate cancer cells treated with rosuvastatin.^147^ One potential mechanism underlying these effects is the suppression of Akt, a regulator of cell adhesion, cytoskeletal remodeling, and epithelial‐mesenchymal transition.^148^ As previously mentioned, statins may target Akt through effects on lipid rafts or other mechanisms.^134^

Cancer stem cells (CSCs) play a crucial role in tumor development and maintenance, and their regulation is closed intertwined with cholesterol control.^149^ It has been documented that cholesterol levels may influence the growth of CSCs, particularly in the small intestine.^150^ In mouse colon cancer model lacking the LPCAT3 enzyme, there is an upregulation of the cholesterol production gene, presumably leading to significantly shorter survival times for the mice.^150^ Studies have also demonstrated that blocking the pathway responsible for cholesterol synthesis may activate the TGF‐β pathway, thereby promoting the differentiation of basal pancreatic cancer cells.^151^ However, it is noteworthy that PDAC patients treated with statins exhibit elevated mesenchymal features, which may have adverse effects.^151^ Further research is essential to fully understand the molecular mechanisms governing the regulation of cholesterol metabolism homeostasis and tumor phenotypic plasticity.

One prospective advantage of statins is their ability to counteract the immune‐suppressive effects induced by high cholesterol levels. Recent preclinical and clinical research across various malignancies have introduced a developing concept wherein statins enhance anti‐tumor immune responses.^152^, ^153^, ^154^, ^155^, ^156^ Dendritic cells (DCs) and other antigen‐presenting cells carry class II major histocompatibility complex (MHC II) molecules, which are raft‐associated proteins crucial for conveying processed tumor antigen peptides to effector cells, thereby triggering anti‐tumor responses.^157^ Elevated cholesterol levels in tumor cells have been shown to suppress the function of DCs.^158^ Tumor cell‐secreted oxysterol further decreases T cell priming and impedes DC migration to lymph nodes.^159^ Cholesterol, through immunological checkpoint activation and CD8+ T cell fatigue, may directly contribute to T cell dysfunction.^160^ Membrane cholesterol interaction with CRAC motifs binding cholesterol also contributes to the stabilization of programmed cell death protein ligand 1 (PD‐L1).^160^ This suggests that reducing cholesterol levels may achieve immune checkpoint blockade immunotherapy and/or interrupt immune checkpoint signaling. Moreover, high cholesterol exposure in the tumor microenvironment is also linked to increased PD‐1 expression and CD8+ T cell exhaustion^118^ through CD8+ T cell infiltration.^161^ Therefore, targeting tumor progression with a cholesterol‐reducing drug like statins can prime DC cells and CD8 + T cells to neutralize tumor cells in conjugation with immune checkpoint inhibitors.

### 3.3 Insights gained from the in vivo studies involving animal models and clinical trials

Statins have been shown to exhibit anti‐cancer effects in‐vivo across various animal models of cancer, complementing their effectiveness in‐vitro. Their efficacy as chemopreventive agents has been established in radiation‐induced mammary tumorigenesis,^177^ chemically‐induced colon tumorigenesis in rodent models,^178^ inoculation of human myeloid leukemia and glioma cancer cells in severe combined immunodeficient mice,^179^, ^180^ and chemically‐induced lung tumor in mice.^181^ Statins have also demonstrated the ability to decrease metastasis in animal mammary tumors,^182^ rat fibrosarcoma,^183^ mouse lymphoma,^184^ murine colon tumors,^185^ and mouse melanoma.^186^ Additionally, statins have been shown to reduce the cardiotoxicity of doxorubicin while simultaneously enhancing its in vivo anticancer efficacy in three tumor models.^187^ Similarly, by blocking tumor‐induced angiogenesis, statins reportedly enhance the anticancer effect of tumor necrosis factor in a mouse model.^188^ In a BALB/c (nu/nu) mice model, statins were found to counteract adriamycin‐induced cancer stem cell characteristics and metastasis in osteosarcoma by downregulating KLF4 and CD133.^189^ Statins have also demonstrated a capacity to lower in‐vivo metastasis in various cancer types. For instance, using a nude mice model, the study revealed that simvastatin dramatically inhibits the development of osteolytic lesions caused by the bone‐metastasizing human A549 lung cancer (LC) cells.^113^ Simvastatin's effect may be attributed to its inhibition of the synthesis and secretion of osteoclastogenic factors by colonized LC cells in the bone.^113^ Simvastatin has been shown to reduce the expression of CD44, a cell surface antigen concentrated in cancer cells that initiate epithelial tumors and spread to other locations.^113^, ^190^ CD44 is known to control the migration and invasion of LC cells. Simvastatin may induce an increase in p53 levels in A549 cells, suppressing CD44 expression and downregulating MMP2 and MMP9.^113^

Numerous population‐based studies have consistently indicated that statins contribute to prolonged survival and improved prognosis in cancer patients. A comprehensive 15‐year large‐scale observational investigation within a Danish subset demonstrated that the use of statins in cancer patients was associated with a reduction in cancer‐related mortality across 13 types of cancers, compared to patients not using statins.^191^ in a meta‐analysis involving 1 111 407 cancer patients, statin use was linked to a remarkable 30% reduction in all‐cause mortality and 40% reduction in cancer‐specific mortality.^192^ A phase II clinical trial evaluated the safety and effectiveness of combining simvastatin and cetuximab in patients with metastatic colorectal cancer who had previously undergone fluoropyrimidine, oxaliplatin, and irinotecan treatments. Among patients with KRAS mutations, only 4 out of 18 (22.2%) were free from progression at the primary end point.^193^ Another study, utilizing a pilot window‐of‐opportunity design, found that preoperative fluvastatin increased prostate cancer cell death, as indicated by an increase in cleaved caspase‐3 without altering intratumoral Ki67, a cancer marker.^194^ This suggests that using fluvastatin prior to radical prostatectomy improves the effect on tumor cells apoptosis. In a phase I clinical trial, rosuvastatin at doses of 1–8 mg/kg/day in conjunction with erlotinib was assessed for safety and dosage of rosuvastatin in patients with advanced solid malignancies.^195^ Although the combination demonstrated an observed disease stabilization rate of 25%, its use is limited due to notable muscle toxicities such as fatigue, muscle weakness, and myalgia.^195^ Nonetheless, findings from a randomized, single‐blind, placebo‐controlled experiment suggest that rosuvastatin can prevent chemotherapy‐induced cardiotoxicity in breast cancer patients.^196^

Overall survival of metastatic renal cell carcinoma (mRCC) patients treated with various anti‐cancer agents including sunitinib, sorafenib, axitinib, temsirolimus, temsirolimus + interferon (IFN)‐α, bevacizumab + temsirolimus, bevacizumab + IFN‐α, or IFN‐α, both with or without statin, was assessed using a pooled analysis treated on phase II and phase III clinical trials. The study compared treatments without statins, which exhibited an overall survival of 18.9 months, with the use of statins in conjunction with other anticancer adjuvants, resulting in an enhanced the overall survival to 25.6 months.^197^ In the context of first‐line chemotherapy for metastatic pancreatic cancer, two phase III clinical trials indicated improved survival rates when statins were included as part of combination therapy.^198^ However, a few clinical investigations have shown ineffectiveness of statin use in certain settings. For example, the combination of pravastatin with cyclosporine A, mitoxantrone, and etoposide in phase I and II clinical trials caused significant toxicity and lacked efficacy.^199^ Furthermore, when used in conjunction with other chemotherapeutic medications, statins did not increase overall or progression‐free survival in patients with prognosis less than 2 years.^199^ Despite these challenges, a substantial body of preclinical and clinical data highlights the synergistic anti‐tumor effects of statins when combined with traditional cancer treatments.^53^ These findings underscore the importance of further investigation to establish the safe and efficient use of statins as adjuvants in cancer treatment.

A recent study involving 303 patients with advanced pancreatic cancer suggests that the use of Simvastatin and atorvastatin is associated with improved overall survival.^200^ Moreover, in patients undergoing radiation, surgery, and chemotherapy for advanced pancreatic cancer, statin therapy demonstrated a notable 2‐year increase in survival, indicating its potential to enhance the effectiveness of advanced pancreatic cancer therapies.^201^ Another study involving 999 colon cancer patients revealed that post‐diagnosis statin use was significantly linked to a lower risk of mortality from all causes and a reduced risk of cancer‐related death.^202^ Similarly, a recent meta‐analysis revealed a significant correlation between statin use and a decline in both overall and CRC‐specific cancer mortality.^203^ Additionally, in a meta‐analysis comprising 59 073 individuals, statin treatment was significantly associated with a lower risk of hepatocellular carcinoma (HCC) progression in liver cancer patients compared to those who did not take statins.^204^ Given the compelling evidence supporting anti‐cancer effect of statins, an increasing number of clinical trials are ongoing to gain insights of using statin as a promising anti‐cancer drug either as monotherapy or combinatorial therapy. Furthermore, we have summarized a list of ongoing clinical trials wherein statins are used in the anti‐cancer therapeutic regime (Table 2).

**TABLE 2:** Summary of ongoing clinical trials using statins in patients with different types of cancers.

ClinicalTrials.gov ID	Statin/combination	Study tittle
NCT06241352	Atorvastatin	Statin Addition to Chemotherapy for Advanced Pancreatic Cancer
NCT03971019	Simvastatin	Survival Benefits of Statins in Breast Cancer Patients (SBSBC)
NCT04601116	Atorvastatin	The MASTER Study (MAmmary Cancer STatin ER Positive Study)
NCT03024684	Atorvastatin	Statin for Preventing Hepatocellular Carcinoma Recurrence After Curative Treatment (SHOT)
NCT04457089	Simvastatin	Statin Therapy to Reduce Progression in Women With Platinum Sensitive Ovarian Cancer
NCT05796973	Atorvastatin	Measuring Oncological Value of Exercise and Statin (MOVES)
NCT03560882	Atorvastatin	A Pilot Trial of Atorvastatin in Tumor Protein 53 (p53)‐Mutant and p53 Wild‐Type Malignancies
NCT03889795	Digoxin+Metformin+Simvastatin	Phase IB Metformin, Digoxin, Simvastatin in Solid Tumors
NCT06030622	Metformin+Simvastatin+Digoxin	Phase 2A Pilot C3 Trial of Recurrent/ Refractory Metastatic Advanced Pancreatic Cancer (C3)
NCT05550415	Simvastatin	The Role of Simvastatin in The Epithelial‐Mesenchymal Transition Process of Breast Cancer
NCT05977738	Pitavastatin calcium	Repurposed Drugs in Research for Cancer Clinical Trials‐ Pitavastatin (ReDiReCCT‐Pita)
NCT04767984	Atorvastatin Calcium	Testing Atorvastatin to Lower Colon Cancer Risk in Longstanding Ulcerative Colitis
NCT04491643	Megestrol Acetate+Rosuvastatin	Megestrol Acetate Plus Rosuvastatin in Young Women With Early Endometrial Carcinoma

### 3.4 Understanding the mechanisms of action of statins

From a mechanistic perspective, the anti‐neoplastic effects of statins have been observed to primarily center around apoptosis,^85^ the inhibition of cell proliferation through STAT3/SKP2 signaling,^84^ and the modulation of the YAP/CD44 growth axis via inactivation of YAP.^205^ Statins have been shown to affect the viability of cancer cells by downregulation of TAZ, which is orchestrated by the transcriptional upregulation of p53.^206^ Additionally, a report has suggested a decrease in survivin levels in APC‐mutated colorectal cells, potentially leading to apoptosis upon statin treatment.^207^ Moreover, Reddy et al. demonstrated that statins can downregulate the chromatin organizer Special AT‐rich binding protein 1 (SATB1) in colorectal cancer cells.^208^ Notably, this effect was observed at protein level only, with no discernible effect at the transcript levels, indicating a regulatory effect at the post‐translational level. SATB1 has been demonstrated to directly contribute to the development and progression of various cancers, including breast cancer^209^ and colorectal cancer.^210^, ^211^ Additionally, SATB1 is a target of the Wnt signaling pathway, and its functional overlap with Wnt signaling is crucial for the tumorigenesis of colorectal cancer.^210^ The SATB family of chromatin organizers is proposed to serve as the master regulators of tumor progression.^211^ SATB1 and SATB2, two closely related members of this family, play distinct roles in various cancer types. Furthermore, survival analysis conducted across an array of cancer types, in correlation with the expression of SATB1 and SATB2, suggests that their tissue‐specific expression contributes to disease prognosis.^211^ It would be intriguing to investigate whether statins also target SATB2. Additionally, it is of significance to delineate the effects of statins on the Wnt signaling pathway. A recent study suggests that statins can induce epigenetic alterations by acting as inhibitors of DNA methyltransferases (DNMT), promoting differentiation in colorectal cancer (CRC) stem cells.^119^ Treatment with statins leads to a significant reduction in the stemness of CRC cells by demethylating the *BMP2* promoter and activating the BMP signaling pathway.^119^ Nevertheless, these novel aspects of statins, which involve targeting the degradation or inhibition of oncogenic proteins, provide fresh insights into the mechanisms of action of statins, which may operate independently of the cholesterol pathway or in conjunction with it.

The FDA recognizes only the oral route of administration for statins. However, there are a few limitations related to administering statins orally. For example, the metabolism in the intestinal wall and the subsequent “first‐pass” metabolism in the liver result in a relatively poor bioavailability of statin medications.^212^ Additionally, factors such as drug permeability, inadequate water solubility, drug efflux pathways, and direct, effective transport to hepatocytes, followed by binding to receptors on the rate‐limiting enzyme for cholesterol biosynthesis, HMG‐CoA reductase, contribute to the constraints on oral bioavailability of statins.^213^, ^214^ To enhance the bioavailability and therapeutic efficacy of statin medications, pharmaceutical scientists have explored novel formulations and alternate methods of administration such as transdermal drug delivery, buccal drug delivery, and intravenous statin among others.^215^

### 3.5 Side‐effects of statin treatment

While statins offer numerous beneficial lipid‐independent pleiotropic effects^216^, ^217^ on the body, such as anti‐thrombotic, anti‐oxidant, and anti‐inflammatory, there are reports indicating the development of statin‐associated muscle symptoms (SAMS) due to statin intolerance. A study revealed that 38% of 7924 outpatients on high‐dose statins experienced muscle pain, hindering their ability to engage in even moderate exertion.^218^ Another study reported that out of 1074 statin‐using patients, 62% of the experienced stiffness, 67% cramps, and 50% noted weakness or a loss of strength during exertion; 42% of patients faced significant disruptions to their daily activities.^219^ A potential mechanism for statin myopathy could be attributed to their role in reducing the prenylation of small GTPase proteins involved in cell growth and maintenance.^220^ Consequently, this leads to decreased formation of ubiquinone (Coenzyme Q10 [CoQ10]). A substantial portion of statin myopathy may be attributed to the depletion of ubiquinone (CoQ10) in the muscles, impairing mitochondrial activity.^220^, ^221^, ^222^, ^223^, ^224^

Furthermore, few studies have explored the effect of statins on the regulation of multidrug resistance proteins, revealing varied susceptibilities to different statins (atorvastatin, simvastatin, and rosuvastatin) in breast cancer cells.^24^ For instance, breast cancer cell lines such as MDA‐MB‐231 and MDA‐MB‐468 exhibit sensitivity, whereas T47D and MCF‐7 cells demonstrate resistance. The resistance in T47D and MCF‐7 cells is attributed to SREBP‐2‐mediated upregulation of HMGCR mRNA and protein expression.^225^ Lovastatin induces autophagy flux and the multidrug resistance proteins MDR1 and TGF‐β1, potentially contributing both resistance and cytotoxicity in breast cancer cells. In vitro studies on hepatocytes indicate that statins, particularly pitavastatin, assist in the excretion of endogenous and exogenous lipophilic substances by modulating MDR2 expression.^226^, ^227^ Additionally, statin treatment induces resistance through an increase in the metabolism of unsaturated fatty acids and cholesterol production, leading to the upregulation of stearoyl‐CoA desaturase (SCD) and HMGCR.^227^ Clinical data further associates polymorphisms in genes, such as MRP2/ABCC2, HMG‐CoA reductase, CETP, TNF‐α, BCRP/ABCG2, P‐gp/ABCB1, MRP1/ABCC1, ApoE, NPC1L1, RHOA, CYP7A1, LDLR and OATP with statin resistance and intolerance.^24^, ^228^

On the contrary, there have been indications that statins may serve as a preventive drug by inhibiting the excessive proliferation of tumor cells fueled by cholesterol or its derivatives.^24^, ^229^, ^230^ Conversely, certain clinical trials have yielded unsatisfactory results when employing statins. For instance, in phase I and II clinical studies, the combination of pravastatin with cyclosporine A, mitoxantrone, and etoposide resulted in significant toxicity and failed to achieve the desired effectiveness.^231^ Furthermore, the overall survival did not show improvement when using statins in conjunction with other chemotherapy drugs.^199^ Consequently, these findings suggest that further investigation is necessary to establish the efficacy and safety of incorporating statins as adjunctive therapy in cancer treatment.

## 4 DISCUSSION

Lipids, cholesterol in particular, are vital components for maintaining cellular homeostasis and facilitating cell division. Tumor progression is characterized by an exploitative utilization of lipid reservoirs, achieved through an aberrant upregulation of the de novo synthesis of cholesterol to support uncontrolled cell proliferation.^232^ In addition, the dysfunctional metabolism of fatty acids in tumor tissues further impairs the membrane lipid composition by altering the ratios of poly‐ and mono‐unsaturated as well as saturated FFAs.^10^, ^11^, ^233^ Furthermore, the tumor microenvironment, which includes immune cells and the extracellular matrix, undergoes reprogramming to meet higher lipid requirements that promote tumor transformation and metastasis.^234^, ^235^ This restructuring results in reduced lipotoxicity and ferroptosis, leading to increased resistance of tumor as well as cancer‐associated stem cells toward therapeutic drugs.

Hence it is crucial to evaluate the lipid status within both the tumor and adjacent tissue when determining the appropriate cancer therapy regime that targets cholesterol‐responsive pathways. The primary and secondary products of the mevalonate pathway are well known to govern processes such as protein synthesis, post‐translational lipid modifications, and signaling components. Targeting the inhibition of this central cascade would not only affect tumor cells but also interfere cholesterol biosynthesis in normal cells, raising concerns about the pleiotropic effects of the drugs. Nevertheless, the effectiveness of drugs like statins as anti‐neoplastic agents is rooted in their mechanism of action. The latest formulations of statins utilized in treatment regimens exhibit greater specificity, improved delivery, and metabolism, resulting in significantly reduced side effects.^25^

From a mechanistic standpoint, statins operate by impeding cholesterol biosynthesis, forcing cells to acquire circulating cholesterol from the bloodstream.^16^ This approach ensures that cellular requirements of cholesterol are met while simultaneously regulating serum cholesterol levels. Nevertheless, the anti‐cancer effects of statins extend beyond cholesterol‐reduction alone. Metabolites within the mevalonate pathway, such as cholesterol derivatives like oxysterols,^236^, ^237^ squalene,^238^ and isoprenoids,^58^, ^239^, ^240^ are recognized contributors to tumorigenesis. Statin treatment inhibits the production of these signaling components thereby curbing tumor progression.^140^, ^241^, ^242^ Farnesylation and Geranylgeranylation are the two isoprenyl modifications that are crucial for signaling, especially in tumor progression.^243^, ^244^ Reports of active role of Rho family of GTPases in tumorigenesis^245^ have initiated the search for their potent inhibitors as cancer therapeutics.^246^, ^247^ It is well‐established that statin‐mediated inhibition of isoprenylation in small GTPases has a direct impact on oncogenic signaling, thereby preventing tumor initiation and making statins one of the candidates.^248^, ^249^, ^250^ A recent report demonstrated metabolic reprogramming in chemo‐resistant small cell lung cancer cells and in vivo xenografts by inhibiting the Mevalonate‐Geranylgeranyl diphosphate pathway using statin.^251^

Several other anti‐neoplastic mechanisms mediated by statins have been reported, targeting the epigenetic machinery of tumor cells independently of their effects on the mevalonate pathway. Studies have proposed that statins play a role in modifying the DNA methylation profile by downregulating DNMT1, thereby altering the transcription profile toward a tumor suppressive phenotype.^252^, ^253^ Another set of findings, focused on colorectal cancer cells, highlighted the significant downregulation of EZH2 histone methyltransferase by statins, particularly when used in combination with HDAC5 inhibitor, leading to an augmented expression of p27.^254^ Statins have also been demonstrated to affect the miRNA levels, consequently modifying the expression of target genes. For example, in hepatocellular carcinoma cells, atorvastatin was observed to upregulate miRNA 145, resulting in the downregulation of PI3K/AKT signaling and promoting cell death.^255^ Studies using simvastatin in colorectal cancer cells and breast cancer cells have revealed an upregulation in miRNAs 192^256^ and 140‐5p,^257^ respectively, initiating cell cycle arrest and apoptosis. Furthermore, an intriguing analysis in triple negative breast cancer (TNBC) cells suggested a mechanism in which fluvastatin treatment inhibited the growth of pre‐neoplastic TNBC cells via miRNA 140‐3p‐1, particularly when combined with aspirin and metformin, aiding in sensitizing resistant cells.^258^

Consequently, recent studies have also focused on the chemo preventive mechanism of statins, with the aim of improved prognosis in the early stages of cancer development.^259^ A meta‐analysis conducted in the context of pancreatic cancer indicated a reduced incidence of tumor development in patients receiving statin therapy.^260^, ^261^ The reports highlighting a positive correlation between statin use and decreased risk of prostate cancer,^262^ biliary tract cancer,^263^ and ovarian cancer^264^ have further strengthened the drug's potential for chemoprevention. Additionally, a significant meta‐analysis study has suggested a moderate decrease in the risk of developing colorectal cancer with statin consumption.^265^ However, It is important to note that many of these studies are observational rather than clinical trials, resulting in heterogeneous data.^266^, ^267^ Another limitation stems from cohort‐specific inferences with limited follow‐up data,^268^ underscoring the necessity for a population‐based analyses and longer‐term patient medical records. Another aspect explored to determine the effect of statins in cancer therapeutics, which could account for the variability in their mode of action across different tissue types, is the effect of genetic polymorphisms in individuals. Following the concept of Mendelian randomization, genetic variations within a population may lead to diverse responses to environmental stimuli, such as the effects of drugs in cancer treatment.^269^ Recently, several reports have suggested a potential cause‐and‐effect relationship between statin use and reduced cancer risk.^270^ These studies highlight the pleiotropic effect of statins, which may be influenced by genetic variations in cholesterol pathway genes, particularly single nucleotide polymorphisms (SNPs) within the HMGCR gene that encodes the enzyme crucial for catalyzing the rate‐limiting step of the MVA pathway.^271^, ^272^, ^273^ One genome‐wide analysis conducted on patients receiving statin therapy identified a polymorphism in the SLCO1B1 gene, encoding a protein responsible for the hepatic uptake of statins, which was associated with an increased risk of myopathy.^274^ Such findings advocate for the genotypic analysis of SNPs within cholesterol metabolism genes that could affect the mode of action of statins in individuals. However, the sensitivity analysis of Mendelian randomization is challenging due to limited data on genetic variations in each cancer type and stage.^275^ A more comprehensive assessment of SNPs is necessary to elucidate the precise mechanism underlying the anti‐neoplastic effects of statins.

In conclusion, statins represent an emerging class of repurposed drugs with potential anti‐neoplastic properties. However, the mechanism underlying their anti‐neoplastic effects and survival benefits remain unclear. Dysregulated cholesterol homeostasis significantly impacts tumor development, yet fundamental questions persist despite our understanding of cholesterol's role in tumorigenesis. To identify targets for cancer prevention and treatment, rigorous in vivo and in vitro experiments are necessary to elucidate the intricate interaction between cholesterol metabolism pathways and cancer‐related processes. Numerous clinical questions regarding the usage of statins in cancer patients remain unanswered, including optimal regimen, tumor types most responsive to statin therapy, and the most effective statins for cancer treatment and prevention. Well‐designed clinical trials and patient‐derived models are needed to fully explore statins' potential as an adjuvant to cancer therapy. Although statins have been the focus of extensive clinical and epidemiological investigations in recent years, the results have been mixed. Hence, carefully planned clinical trials are essential to confirm their anti‐cancer effects. While statins alone may not suffice as cancer monotherapy due to insufficient clinical data, a number of preclinical and clinical studies suggest that combining statins with other cancer therapies targeting various molecular signaling pathways can enhance their efficacy. These include pathways related to cell cycle and apoptosis, epigenetic modifiers such as HDACs and DNMTs, metabolic alterations focused on mevalonate pathway, and the targeting of protein farnesylation and geranylgeranylation. Predictive biomarkers of statin sensitivity are crucial for patient stratification, as not all cancers respond to statin‐mediated suppression of the mevalonate pathway. Precision medicine advancements offer promise in personalized medicine by identifying biomarkers that predict statin responsiveness and tailoring statin treatment based on individual genotypic profiles.

Future research endeavors should prioritize conducting large‐scale phase III randomized clinical trials involving cancer patients and utilizing patient‐derived models. These trials are crucial for validating promising predictive biomarkers and elucidating the precise role of statins in cancer treatment and prevention. Such studies will help confirm the specificity of statins' anti‐cancer effects and shed light on the precise molecular mechanisms underlying their tumor‐suppressive properties. These clinical trials should encompass investigations into both standalone statin treatments and their use in combination with proposed inhibitors. It is worth noting that hydrophobic and hydrophilic statins follow distinct metabolic routes, leading to differences in their cellular bioavailability after ingestion, a phenomenon termed as pharmacokinetics.^276^ However, when statins are incorporated into combinatorial therapies alongside other repurposed inhibitors, it could potentially alter the bioavailability of both compounds.^277^ By conducting these clinical trials would can obtain a comprehensive understanding of the dynamics of these drug combinations in the context of cancer therapeutics.

## AUTHOR CONTRIBUTIONS

**Sneha Tripathi:** Conceptualization; writing – original draft; writing – review and editing. **Ekta Gupta:** Conceptualization; writing – original draft; writing – review and editing. **Sanjeev Galande:** Conceptualization; funding acquisition; writing – original draft; writing – review and editing; supervision.

## FUNDING INFORMATION

The work was supported by research grant from Department of Biotechnology (DBT), Government of India to SG (BT/ATGC/127/SP39484/2020). SG is also a recipient of the JC Bose Fellowship (JCB/2019/000013) by the Science and Engineering Research Board, Government of India.

## CONFLICT OF INTEREST STATEMENT

The authors declare that they have no competing interests.

## ETHICS STATEMENT

Not applicable.

## Data Availability

Data sharing is not applicable to this article as no new data were created or analyzed in this study.
